# Portal vein stent placement for the treatment of postoperative portal vein stenosis: long-term success and factor associated with stent failure

**DOI:** 10.1186/s12893-017-0209-y

**Published:** 2017-02-01

**Authors:** Atsushi Kato, Hiroaki Shimizu, Masayuki Ohtsuka, Hideyuki Yoshitomi, Katsunori Furukawa, Masaru Miyazaki

**Affiliations:** Department of General Surgery, Chiba University Graduate School of Medicine, 1-8-1 Inohana, Chuo-ku, Chiba 260-8670 Japan

**Keywords:** Portal vein stent placement, Hepatobiliary pancreatic surgery, Portal vein stenosis

## Abstract

**Background:**

Portal vein stenosis develops due to different causes including postoperative inflammation and oncological processes. However, limited effective therapy is available for portal vein stenosis. The objectives of this study were to evaluate the efficacy of a portal vein stent for portal vein stenosis after hepatobiliary pancreatic surgery and to determine the factors associated with stent patency.

**Methods:**

From December 2003 to December 2015, portal vein stents were implanted in 29 patients who had portal vein stenosis after hepatobiliary pancreatic surgery. We conducted a retrospective analysis to evaluate the efficacy and safety of portal vein stent placement. Twelve clinical variables were analyzed for their role in stent patency.

**Results:**

The symptoms before portal vein stent placements included nine patients with hepatic encephalopathy, six patients with gastrointestinal bleeding, four patients with ascites, and four patients with hyperbilirubinemia. Portal vein thrombosis due to postoperative portal stenosis was found in four patients. Portal vein stent were successfully implanted without any major complications. Of the 21 patients with symptoms, 17 showed improvement, and stent patency was maintained in 22 (76%) patients. The presence of a collateral vein is the only variable related to the development of an occlusion after portal stenting.

**Conclusion:**

Portal vein stent were implanted safely and had good long-term patency. This procedure is useful to relieve portal hypertension-related symptoms and to improve the quality of life. Our data strongly suggest that embolization to block blood flow in a collateral vein during portal vein stent placement will improve the patency of the stent.

## Background

Portal vein stenosis develops due to various causes, including both postoperative inflammation and oncological processes. Benign portal vein stenosis is a complication associated with portal vein reconstruction for liver transplantation and after hepatobiliary pancreatic surgery. It is also caused by inflammation surrounding the portal vein, such as pancreatitis [1–3]. In addition, the incidence of portal vein stenosis due to hepatobiliary pancreatic malignancies, including hepatocellular carcinoma, pancreatic cancer, and biliary tract cancer, is 15 to 24% of the cases of extraheptic portal vein stenosis [4–6]. Furthermore, portal vein stenosis is likely to develop due to local recurrence after hepatobiliary pancreatic surgery [7, 8]. Portal vein stenosis is associated with portal hypertension and with the development of varices in the esophagus, stomach, duodenum, and small bowel. These varices frequently cause refractory gastrointestinal (GI) bleeding [9, 10]. Portal hypertension is also a cause of liver dysfunction associated with both abdominal ascites and hepatic encephalopathy.

Limited effective therapy is available for portal vein stenosis. A recent study of the clinical efficacy of portal vein stent placement in patients with either malignant tumors or benign portal stenosis showed that portal hypertension was improved, and clinical symptoms were resolved [11, 12]. On the other hand, thrombus and/or tumor growth sometimes cause re-occlusion, and the stent patency rate is 60–100% [13–15].

The objectives of this study were to evaluate the efficacy of portal vein stent placement for portal vein stenosis after hepatobiliary pancreatic surgery and to identify the factors involved in stent patency.

## Methods

### Patients

From December 2003 to December 2015, portal vein stents were implanted at a single institution in 29 patients (21 males and 8 females, mean age 65.9 ± 10.0 years, range 38–83 years) who had portal vein stenosis after hepatobiliary pancreatic surgery. We retrospectively reviewed the clinical data of these 29 patients.

Postoperative pathological diagnosis showed intrahepatic cholangiocarcinoma in three patients, extrahepatic cholangiocarcinoma in 13, gallbladder cancer in 1, pancreatic cancer in 8, intraductal papillary mucinous neoplasms in 2, autoimmune hepatitis in 1, and Budd-Chiari syndrome in 1. In terms of surgical procedures, liver resection was performed in 15 patients (live donor liver transplant in two patients), and pancreatic resection was performed in 14 patients. Portal vein resection and reconstruction were performed in 14 patients. The clinical characteristics of these patients are shown in Table 1.

**Table 1:** Characteristics of patients (*n* = 29) with portal vein stent placement

Age (mean year ± SD)	65.9 ± 10.0
Sex (mail: female)	21: 8
Pathological Diagnosis	
Biliary Tract Cancer	17
Pancreas Cancer	8
Intraductal Mucinous Papillary Neoplasms	2
Autoimmune Pancreatitis	1
Budd Chiarri Syndrome	1
Surgical Procedure	
Liver Resection	15
Pancreas Resection	14
Etiology	
Tumor Recurrence	19
Vascular Orientated	10
Involved Vessels	
Portal Vein	16
Superior Mesenteric Vein - Portal Vein	13
Lesion Length (mm)	29.1 ± 22.0
Collateral Vein	
Yes	17
No	12
Stenosis	
Complete	16
Incomplete	13
Anticoagulation (days)	1.75 ± 2.34
PV pressure (mm H_2_O)	35.2 ± 6.2
Stent Patency (days)	519 ± 643

Ten patients with vascular-orientated benign stenosis developed portal vein stenosis, and the remaining 19 patients had portal vein stenosis due to tumor recurrence. All of the patients were diagnosed with portal vein stenosis by enhanced CT. Portal vein stenosis was localized in the region extending from the superior mesenteric vein (SMV) to the portal vein (PV) in 13 patients, and the main portal vein was stenosed in 16 patients. Four patients, who had developed stenosis early after surgery due to portal vein thrombosis, underwent surgical thrombectomy and stenting in the portal vein via the ileocolic vein during surgery. In three patients, who had developed portal vein stenosis after surgery, we tried to reconstruct the portal vein. However, we could not detach the severe adhesion to the portal vein and performed portal vein stent placement via the ileocolic vein during surgery.

### Symptoms

The major symptom found in nine patients was hepatic encephalopathy. GI bleeding was found in six patients, ascites was found in four patients, and hyperbilirubinemia was found in two patients. Portal vein thrombosis due to postoperative portal stenosis was found in four patients. The remaining four patients had no symptoms. These asymptomatic patients were as follows: one patient who underwent a live donor liver transplant had marked stenosis in the anastomotic site of the portal vein and underwent portal vein stent placement to prevent portal vein occlusion. Three patients receive a portal vein stent, including one patient with portal vein stenosis due to postoperative inflammation and two patients with portal vein stenosis caused by a recurrent tumor.

### Stent placement

Informed consent was obtained from all of the patients and their families prior to portal vein stent placement. The portal vein stents were implanted by two procedures. The procedures included ultrasonography (US)-guided percutaneous transhepatic puncture of the intrahepatic portal vein and a surgical approach for insertion via the ileocolic vein. A 5-French sheath was inserted into the portal vein, and portography using a catheter was performed in both procedures. After that, the length of the stenotic segment was determined, and a self-expandable metallic stent was placed in the stenotic region. When selecting a stent, it is ideal to choose a stent that is approximately 1 cm longer at both ends of the collapse. A collateral vein was found in 17 patients. However, the blood flow in the collateral vein immediately disappeared after stenting, and no embolization of the collateral vein was performed. Patients who had portal vein stenosis after stenting the portal vein due to insufficient expansion of the stent underwent balloon dilation in the stenotic area.

Anticoagulation therapy with heparin sodium was used for all patients within 0–7 days after portal vein stent placement. Subsequently, warfarin potassium, an oral anticoagulant, was administered to maintain the international normalized ratio of prothrombin time (PT-INR) at 1.5–2.0 for at least 1 year.

### Follow-up

Complications with the stent placement were evaluated. Stent patency was evaluated at intervals of 2–3 months for the first year and at intervals of 3–6 months thereafter by measuring the blood flow in the stent using either Doppler US or contrast-enhanced CT. The blood flow in the stent was evaluated immediately if portal hypertension developed clinically. The patency period was defined as the time from stent placement to stent occlusion. The mean follow-up period for all patients was 19.1 ± 24.9 months.

Twelve variables, including age, sex, pathological diagnosis, surgical procedures, portal vein combined resection, etiology, involved vessels, lesion length, presence of a collateral vein, type of stent procedure, time of starting anticoagulation therapy, and portal vein pressure before stenting, were used to confirm whether these variables were associated with stent patency.

#### Data analyses

Differences between the matched groups were evaluated using the Fisher’s exact test for paired proportions for categorical variables. The overall stent patency was calculated according to the Kaplan–Meier method. Statistical significance was defined by *p* values less than 0.05.

## Results

Portal vein stent placement was successfully performed, and blood flow in the portal vein was confirmed in all patients. No serious complications, excluding a minor complication (transient fever) in three patients, were observed after portal vein stent placement. Furthermore, no hemorrhagic complications due to anticoagulation therapy after stenting were found.

Stent placement was performed by a percutaneous transhepatic approach in 22 patients and by a laparotomy via the transileocolic vein in seven patients. Four of the seven patients who underwent stent placement via the transileocolic vein had acute portal vein thrombosis, and a stent was placed in a stenotic region after thrombectomy. Two patients, who were found to have portal vein stenosis after a live donor liver transplant, were unable to have re-anastomosis of the portal vein. Therefore, stent placement via the transileocolic vein was performed.

**Fig. 1:**
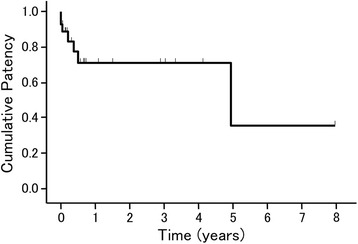
Kaplan-Meier cumulative portal vein stent patency in 29 patients

Stent patency was maintained in 22 (76%) of the 29 patients during the observation period, and the mean stent patency period in all patients was 17.3 ± 21.4 months (1–2318 days) (Fig. 1). Stent occlusion was found in seven patients, including three with acute stent occlusion due to thrombosis, in one patient with stent occlusion due to thrombosis at 80 days after stenting, and in three patients with stent occlusion due to tumor growth at 145 to 1827 days after stenting. No further intervention including thrombectomy or re-stentig was performed in patients with portal stent occlusion because of general poor condition. Of the 21 patients with symptoms, 17 had improved symptoms after the stent placement. Two patients, who had hepatic encephalopathy with acute stent occlusion due to thrombosis early after stenting, did not improve. These two patients with acute stent occlusion were followed up without re-stenting, and the symptoms gradually resolved in one of the patients via development of a collateral vein. For the other patient, the symptoms were relieved by conservative therapy until the patient died due to cancer recurrence approximately 9 months later. One patient with ascites and one patient with hyperbilirubinemia did not improve after stenting.

**Fig. 2:**
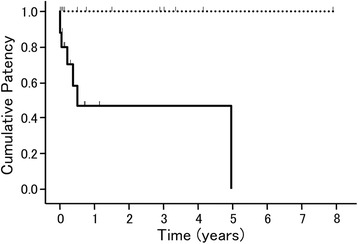
Kaplan-Meier cumulative portal vein stent patency. The solid line shows the cases with a collateral vein, and the dotted line shows the cases without a collateral vein

The relationship between the 12 variables and stent patency was evaluated (Table 2). The presence of a collateral vein was the only variable associated with the development of stent occlusion (Fig. 2). Factors related to acute stent occlusion were evaluated but no statistically significant factor was identified.

**Table 2:** Portal venous stent patency in patients (*n* = 29) following postoperative portal vein stenosis

Variables		Patency		*p* value
		Yes	No	
Age	≤69 years old	10	5	
	>69 years old	12	2	0.2310
Sex	Male	17	4	
	Female	5	3	0.2993
Pathological Diagnosis	Benign	2	0	
	Malignant	20	7	0.4084
Surgical Procedure	Liver Resection	11	4	
	Pancreas Resection	11	3	0.7419
Portal Vein Combined Resection	Yes	11	3	
	No	11	4	0.7419
Etiology	Tumor Recurrence	14	5	
	Vascular Orientated	8	2	0.7056
Involved Vessels	PV	13	3	
	SMV-PV	9	4	0.4519
Lesion Length	≤16 mm	10	5	
	>16 mm	12	2	0.2310
Collateral Vein	Yes	10	7	
	No	12	0	0.0107^a^
Stent Procedures	Transhepatic	15	7	
	Transileocolic	7	0	0.0866
Anticoagulation Therapy	≤1 day	17	6	
	>2 day	4	1	0.7757
PV pressure	≤33.5 mmH_2_O	10	1	
	>33.5 mmH_2_O	7	3	0.2230

*PV* portal vein, *SMV* superior mesenteric vein

^a^Denotes statistical significance

## Discussion

Postoperative portal vein stenosis is a surgical complication seen either after anastomosis of the portal vein or inflection of the portal vein during hepatectomy, pancreatectomy, and liver transplantation. Consequently, portal vein occlusion is frequently complicated by thrombosis. Since portal vein stenosis is likely to occur early after surgery, it is necessary to monitor the portal flow using color Doppler US [16, 17]. On the other hand, patients who developed local recurrence surrounding the portal vein after radical resection for hepatobiliary pancreatic cancer often have portal vein stenosis.

Many patients have portal hypertension due to both postoperative portal vein stenosis and occlusion, and these patients also develop refractory ascites, GI bleeding, and hepatic encephalopathy associated with elevated blood ammonia caused by the portal-systemic shunt. Postoperative portal vein stenosis not only increases morbidity and mortality but also has an effect on the long-term quality of life (QOL).

Four of our patients with portal vein stenosis that developed early after surgery had complicated portal vein thrombosis, a thrombectomy, and then a portal vein stent placement that successfully perfused blood flow in the portal vein. Refractory ascites associated with portal hypertension was found in four patients, GI bleeding in six patients, hepatic encephalopathy in nine patients, and hyperbilirubinemia in two patients. All of these patients underwent portal vein stent placement. The symptoms improved in 17 of the 21 patients (81%). Thereafter, the patients had a good QOL. Serious complications after portal vein stent placement, including hepatic artery aneurysm and liver abscess, have been reported with the use of the hemoperitoneum for the percutaneous transhepatic approach and with the use of the ileus for the transileocolic approach [11, 15]. However, the incidence of serious complications in those studies was low. In fact, no serious complications were observed after portal vein stent placement in our study. Thus, these procedures were performed in an extremely safe manner with a low incidence of complications.

There are several studies on the long-term durability of portal vein stent placement. Tsukamoto et al. showed that the stent patency rate was 100% for the observation period of 27.9 ± 10.7 months in seven patients with malignant portal vein stenosis [13]. Ko et al. reported that the stent patency rate was 78% for the observation period of 66.6 ± 16.1 months in nine patients with portal vein stenosis after a live donor liver transplant [14]. Kim et al. indicated an 89% stent patency rate for the observation period of 23.5 ± 22.5 months in 19 patients with curative surgery for pancreatic and biliary neoplasms [11]. Zhou et al. conducted portal vein stent placement in patients with hepatobiliary pancreatic cancer and showed a 75% stent patency rate for the observation period of 13 ± 11 months [15]. We also confirmed relatively good stent patency (76%) for the observation period of 17.3 ± 21.4 months. As described above, several studies showed relatively good stent patency for a long-term observation period, however, there are few studies that have analyzed the factors associated with the patency of a portal vein stent.

Yamakado et al. showed that the stent patency rate was 60% for the observation period of 11.9 ± 12.9 months in 40 patients with malignant portal vein obstruction [6]. They conducted multivariate analysis and identified three factors associated with stent occlusion, including splanchnic vein involvement, cirrhotic patients classified as Child-Pugh class C, and obstruction of the portal vein system. We analyzed 12 variables associated with stent patency. The presence of a collateral vein was the only variable related to the development of stent occlusion in our study. This result suggested that decreased blood flow in the portal vein was related to stent occlusion. These results strongly suggested that embolization should be performed to block blood flow in a collateral vein in patients who develop a collateral vein. Recently developed metallic stents are highly flexible and expandable, and thus the length and diameter are adjustable for placement of an appropriate sized stent in a stenotic region. Therefore, stents can be used for long portal vein stenosis even in the splanchnic vein, and this may contribute to long-term patency.

Another factor for stent patency is anticoagulant therapy to prevent stent-related thrombosis. Many physicians use anticoagulant therapy [15, 18]. On the other hand, Novellas et al. showed that the stent patency rate was 79% in 11 patients without anticoagulation therapy [12]. Kim et al. indicated the risks of anticoagulant therapy, including bleeding from the percutaneous transhepatic route, GI bleeding, and heparin-induced thrombocytopenia [11]. Stent occlusion associated with acute thrombus formation after stent placement is an important issue, and acute thrombosis can be prevented by anticoagulant therapy as soon as possible after stent placement. Therefore, we treated all of our patients with anticoagulant therapy. No hemorrhagic complication was found in this study. However, acute stent occlusion due to thrombus formation was found in two patients who underwent anticoagulant therapy with heparin on the day of stent placement. Further prospective studies should be conducted to examine the efficacy of anticoagulant therapy.

## Conclusion

Portal vein stent placement for patients with portal vein stenosis after hepatobiliary pancreatic surgery is performed safely and has good long-term patency. Our data strongly recommend embolization to block blood flow in a collateral vein during portal vein stent placement, because the presence of a collateral vein is the only variable related to the development of stent occlusion.

## Abbreviations

GI: Gastrointestinal
PT-INR: The international normalized ratio of prothrombin time
PV: Portal vein
QOL: Quality of life
SMV: Superior mesenteric vein
US: Ultrasonography
